# Mutations in Acetylcholinesterase2 (*ace*2) increase the insensitivity of acetylcholinesterase to fosthiazate in the root-knot nematode *Meloidogyne incognita*

**DOI:** 10.1038/srep38102

**Published:** 2016-11-29

**Authors:** Wen-Kun Huang, Qin-Song Wu, Huan Peng, Ling-An Kong, Shi-Ming Liu, Hua-Qun Yin, Ru-Qiang Cui, Li-Ping Zhan, Jiang-Kuan Cui, De-Liang Peng

**Affiliations:** 1 State Key Laboratory for Biology of Plant Diseases and Insect Pests, Institute of Plant Protection, Chinese Academy of Agricultural Sciences, Beijing, 100193, China; 2 School of Minerals Processing and Bioengineering, Central South University, Changsha, 410083, China; 3 Key Laboratory of Biometallurgy of Ministry of Education, Central South University, Changsha, 410083, China; 4 School of Agricultural Sciences, Jiangxi Agricultural University, Nanchang, 330045, China.

## Abstract

The root-knot nematode *Meloidogyne incognita* causes severe damage to continuously cropping vegetables. The control of this nematode relies heavily on organophosphate nematicides in China. Here, we described resistance to the organophosphate nematicide fosthiazate in a greenhouse-collected resistant population (RP) and a laboratory susceptible population (SP) of *M. incognita*. Fosthiazate was 2.74-fold less toxic to nematodes from RP than that from SP. Quantitative real-time PCR revealed that the acetylcholinesterase2 (*ace*2) transcription level in the RP was significantly higher than that in the SP. Eighteen nonsynonymous amino acid differences in *ace2* were observed between the cDNA fragments of the RP and SP. The acetylcholinesterase (AChE) protein activity in the RP was significantly reduced compared with that in the SP. After knocking down the *ace*2 gene, the *ace*2 transcription level was significantly decreased, but no negative impact on the infection of juveniles was observed. The 50% lethal concentration of the RNAi RP population decreased 40%, but the inhibition rate of fosthiazate against AChE activity was significantly increased in RP population. Thus, the increased fosthiazate insensitivity in the *M. incognita* resistant population was strongly associated with mutations in *ace*2. These results provide valuable insights into the resistance mechanism of root-knot nematode to organophosphate nematicides.

The root-knot nematode (RKN) *Meloidogyne incognita* is widely distributed in soils cultivated with vegetables, causing losses of up to $400 million annually^1^. With the continuous cropping of high-production-value vegetables in the greenhouse, the economic damage of vegetables caused by RKNs is continuously increasing, despite vigorous control efforts. Many physical and chemical methods, such as fumigation of infected soils^2^ and amendment or biofumigation with crop residues^3^, have been attempted to control RKNs. Some biological agents, such as avermectins^1^,^4^, *Paecilomyces lilacinus*^5^, *Bacillus methylotrophicus*^6^, and *Pochonia chlamydosporia*, have also been used to control RKNs with high dosage^7^,^8^. However, these biological agents are expensive, limiting the feasibility of their extensive use^9^. Therefore, high-efficacy but low-cost nematicides, such as organophosphates (OPs) and carbamates (CBs), have been widely used as alternatives to control RKNs^3^,^10^,^11^.

Fosthiazate is one of the most widely used OP nematicides in northern China, which has wide spectrum efficacy against both nematodes and arthropods. The toxicity of OP nematicides reflects the inhibition of acetylcholinesterase (AChE, EC 3.1.1.7), an enzyme that terminates nerve impulses by hydrolysing acetylcholine (ACh) to acetic acid and choline at the synaptic terminal and neuromuscular junction in most vertebrates, insects and nematodes^12^. The extensive use of OP nematicides has resulted in the rapid development of pesticide resistance in arthropods. Many previous studies have demonstrated increased metabolic detoxification, consistently associated with the resistance to the two OPs malathion and acephate in both field populations and laboratory-selected populations of the plant bug *Lygus lineolaris*^13^,^14^,^15^. Increased esterase activity and insensitive AChE were involved in the resistance to the OP insecticide trichlorfon in a field population of *L. hesperus*^13^,^16^. OP-insensitive AChE was involved in chlorpyrifos resistance in various populations of the plant bug *Apolygus lucorum* and the predatory mite *Kampimodromus aberrans*^17^,^18^,^19^. In addition, mutations in at least 18 *Caenorhabditis elegans* genes were shown to confer resistance to the OP-like carbamate pesticide aldicarb^20^. Alterations of gene expression in *C. elegans* were found to be associated with organophosphate pesticide intoxication and recovery^21^. Class C of acetylcholinesterase of *Meloidogyne* had high affinity for acetylcholine, but was highly resistant to carbamate and organophosphate inhibitors^22^,^23^. However, little is known about the resistance level of RKNs to the OP nematicide fosthiazate, particularly the underlying molecular mechanism of resistance.

Here, we present the results of an investigation of a greenhouse population of *M. incognita* from China showing low-level resistance to the OP nematicide fosthiazate and provide evidence of the underlying resistance mechanism. AChE cDNAs, acetylcholinesterase-1 (*ace*1) and acetylcholinesterase-2 (*ace*2), were cloned and sequenced from fosthiazate-susceptible (SP) population and fosthiazate-resistant (RP) populations of *M. incognita*. Bioassays and RNA interference (RNAi) data indicated that this resistance largely reflected increased AChE insensitivity, and mutations in the *ace*2 gene were associated with resistance to fosthiazate in *M. incognita*.

## Results

### Mutations in *ace*2, the cDNA encoding AChE

**Figure 1:**
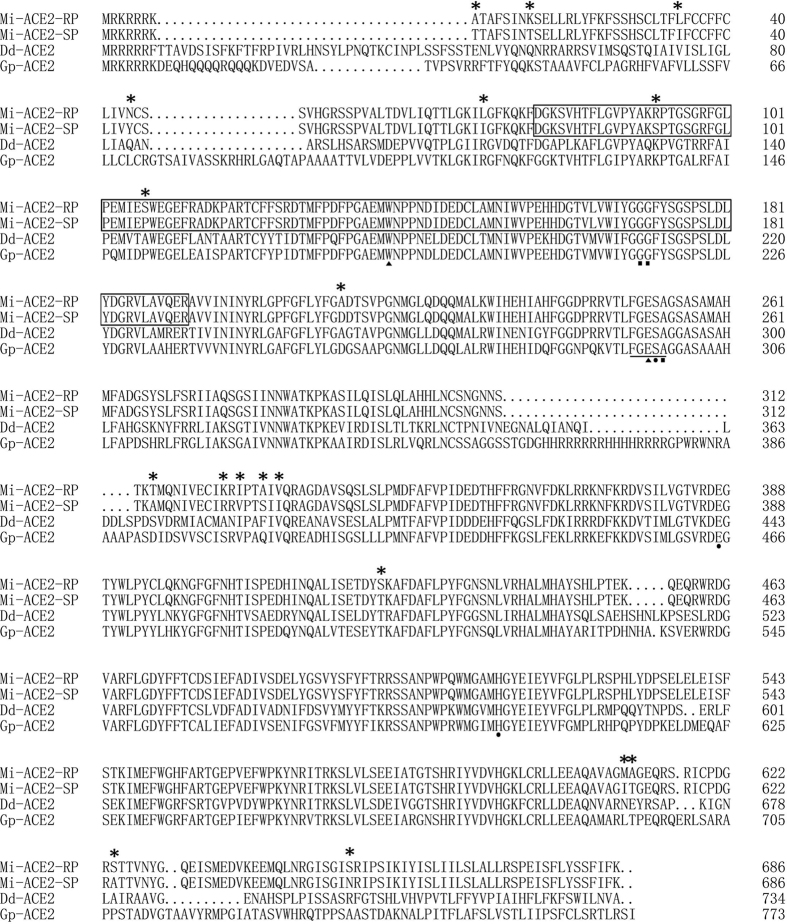
Alignment of the AChE2 amino acid sequences from susceptible (SP) and resistant (RP) populations of *M. incognita*. The mutation sites are marked with asterisks. The catalytic triad residues, oxyanion hole and choline-binding site are respectively indicated with dots, solid triangles and quadrangles. The fragments used in RNAi are boxed. Amino acid sequences from ACE2 of *Ditylenchus destructor* and and *Globodera pallida* are used as homologous sequences.

Sequences of *ace*1 and *ace*2 in both resistant and susceptible populations of *M. incognita* were cloned and submitted to GenBank. Based on sequence analyses, the *ace*1 coding region contained 1971 bases and encoded 656 amino acid residues, with 100% amino acid sequence identity in the cDNAs of the RP and SP populations (GenBank accession number: KU366258). The *ace*2 coding region contained 2061 bases and encoded 686 residues, with 97.4% amino acid sequence identity in the cDNAs of the RP (GenBank accession number: KU360593) and SP populations (GenBank accession number: KU366259). Both sets of *ace*2 sequences encoded most of the key motifs required for catalytic function, such as the conserved aromatic residues, intrachain disulphide bonds, the catalytic triad, the nucleophilic elbow, and choline-binding sites (Fig. 1). To identify the mutation of *ace*2, 30 clones were sequenced from RP and SP populations, respectively, and 68 nucleotide acid sequence differences were observed in 6 clones between the two fragments of the RP and SP populations. The amino acid sequences deduced from the nucleotide acid sequences revealed 18 nonsynonymous differences between the RP and SP populations. However, no differences were observed between the catalytic triad residues, oxyanion holes and choline-binding sites in the RP and SP populations.

### RNAi of *ace*2 did not affect the infection of *M. incognita*

To investigate the relative importance of *ace*2 for nematode infectivity, the *ace*2 gene of *M. incognita* was knocked down using *in vitro* RNAi. The nematodes were soaked in dsRNA for 6 h and then inoculated onto tomato roots. RNAi of *ace*2 had no negative impact on the infection of juveniles compared with non-dsRNA-soaked juveniles (Table 1, Table 2). There were no significant differences between the infections of RNAi-egfp (an reporter gene, enhanced green fluorescent protein) treated juveniles and non-dsRNA-treated juveniles.

**Figure 2:**
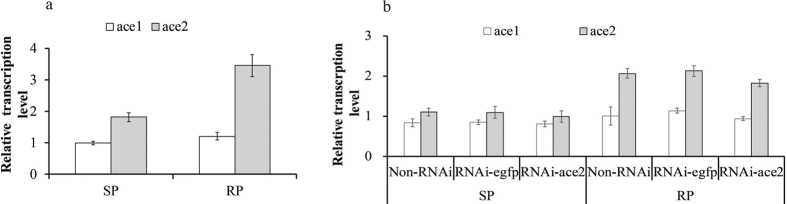
Relative *ace*2 expression levels in susceptible (SP) and resistant (RP) populations of *M. incognita.* (**a**) Transcription levels in RP and SP populations prior to *ace*2 gene interference using dsRNA. (**b**) Transcription levels in RP populations after the *ace*2 gene was subjected to interference using dsRNA. The transcription level of RNAi-egfp in the RP population was used as a control.

RP population increased the *ace*2 transcription level. The transcription levels of the non-RNAi, RNAi-ace2 and RNAi-egfp populations were determined using quantitative RT-PCR. The control actin gene expression levels were adjusted to the same level in different populations prior to qRT-PCR. Prior to *ace*2 gene knockdown, the *ace*2 transcription level in the RP population was significantly higher than that in the SP population (P ≤ 0.05). In addition, the relative *ace*2 expression level was higher than that of *ace*1 in the RP population (Fig. 2a). After the *ace*2 gene was knocked down, the *ace2* transcription level in the RNAi-ace2 population was 11.6% lower than that in the non-RNAi RP (Fig. 2b). However, no significant difference was observed between RNAi-ace2 and non-RNAi treated juveniles of RP and SP populations. No significant difference was observed between the RNAi-egfp and non-RNAi juveniles both in RP and SP populations.

### The *M. incognita* RP shows minor resistance to the nematicide fosthiazate

Compared with the SP population, the RP population developed 2.74-fold resistance to fosthiazate after seven years of greenhouse selection (Table 3). However, no development of resistance to fenamiphos and phonamiphos was observed in the RP populations. Neither fenamiphos nor phonamiphos showed significantly different toxicity levels between the RP and SP populations.

After the *ace*2 gene knockdown, the RNAi RP population was more sensitive to fosthiazate than the non-RNAi RP population. The LC_50_ of the RNAi RP population decreased 40% compared with the non-RNAi RP population (Table 4). However, no significant difference was observed in RNAi SP and non-RNAi SP population. No significant difference was observed between the non-RNAi and RNAi-egfp populations.

### AChE enzyme activity was decreased in the RP population

To examine the enzyme activity of AChE in different populations, different substrates were used to analyse the sensitivity of AChE. RP population showed a 13% reduction AChE activity compared to SP population. However, the activity levels of non-specific esterase and oxidase enzymes did not significantly differ between the RP and SP populations (Table 5).

### The RP population increased the insensitivity of AChE to fosthiazate

**Figure 3:**
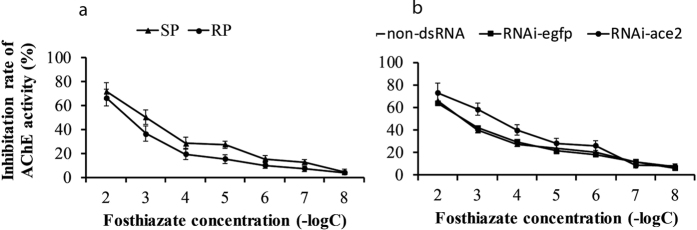
Insensitivity of AChE to fosthiazate in RP and SP populations of *M. incognita*. (**a**) Insensitivity of AChE to fosthiazate in RP and SP populations prior to *ace*2 gene interference using dsRNA. (**b**) Insensitivity of AChE to fosthiazate in RP populations after *ace*2 gene interference using dsRNA. The inhibition rates of different fosthiazate concentrations against AChE were analysed using the minus logarithm of the concentration (−logC).

To detect the relationship between the inhibitory activity of fosthiazate and the AChE, the *ace*2 gene was knocked down with dsRNA, and the fosthiazate inhibition rate was measured after RNA interference. Prior to silencing the *ace*2 gene in the RP population, the inhibition rate of fosthiazate against AChE activity in the RP population was significantly lower than that in the SP population (10^−7^ vs. 10^−2^ M) (P ≤ 0.05) (Fig. 3a). After the *ace*2 gene was knocked down, the inhibition rates of fosthiazate against AChE activity in the dsRNA-interference populations were significantly higher than those in the non-dsRNA-interference populations (10^−6^ vs. 10^−2^ M) (Fig. 3b). No significant difference in the inhibition rate was observed between the non-dsRNA and RNAi-egfp populations.

## Discussion

Resistance to fosthiazate can be a serious problem in the RKN *M. incognita* because OP nematicides are effective control agents that are frequently and regularly used in greenhouse crops. Based on the results of the present study, the resistance of *M. incognita* to fosthiazate has been circumstantiated in tomato greenhouse, where chemical pest control strategies have been used for at least 7 years; however, the underlying molecular mechanism remains poorly understood. The fosthiazate-resistant population exhibited lower levels of AChE activity than the susceptible population, suggesting that a modified AChE might contribute to the reduced substrate affinity^17^,^21^. RNAi results further confirmed that the *ace*2 gene plays a major role in the interaction of AChE and fosthiazate, although mutation of *ace*2 did not affect nematode infection.

The amino acid changes in AChE are found to confer target site resistance in many species^24^,^25^. General carbamate resistance in several mosquitos is due to the substitution in the oxyanion hole. Substitutions adjacent the oxyanion hole in an aphid species and various motifs around the active site in three fly species are implicated in OP resistance. In the herbivorous mite *Tetranychus urticae*, resistance to OPs reflects modifications and insensitivity to AChE enzymes harbouring the amino acid substitution F331W^17^. The chlorpyrifos resistance of the plant bug *A. lucorum* is strongly associated with serine-substituted AChE. Target site resistances as a result of modified AChE conferring high levels of insensitivity to OP and CB have also been described in predatory mites and plant bugs^17^,^19^. In RKN *M. incognita* and *M. arenaria*, the presence of significant amounts of class C AChE have been associated with the resistance mechanisms of OPs and CB^26^. In *B. xylophilus*, Kang *et al*.^27^ proposed that soluble AChE plays a key role in the chemical defence systems of nematodes against various xenobiotics. Thereafter, the high threshold of insensitivity to fosthiazate-resistant populations of *M. incognita* might reflect either a modified form of AChE that is resistant to inhibitors or the differentially biochemical sensitivity of this enzyme. To our knowledge, this study represents the most comprehensive evaluation of the potential OP resistance mechanisms in the RKN to date. However, the actual amino acid alterations in AChE result in a property change in present study remain to be discovered.

Full sequencing of *ace*2 revealed that the *M. incognita* RP differed from the SP in terms of non-synonymous mutations that introduced several amino acid substitutions into the AChE open reading frame. The area of mutation in the AChE catalytic site is a part of the oxyanion hole, a functional domain required for AChE activity^28^. In the *Bractocera oleae* population and several *Drosophila* populations^29^, substitutions of amino acids I to V have been associated with OP resistance^30^. In the present study, the *ace*2 sequences obtained includes residues associated with substitution of OP and/or carbamates resistance in the AChEs of other species^25^. However, although 18 nonsynonymous differences were observed in *ace*2, the target site resistance of these mutated amino acid residues has not been demonstrated in *M. incognita*.

According to the inhibition activity analysis, the AChE of *M. incognita* was inhibited by the OP nematicide fosthiazate, a widely known powerful anti-AChE agent against many mammals and insects^9^. Because both ACE1 and ACE2 have been proposed as the main post-synaptic ACE in plant parasitic nematodes, the strong inhibition of these post-synaptic enzymes is likely to result in high toxicity to nematicides. In the analyses of the inhibitory properties of the three AChEs of the pinewood nematode *Bursaphelenchus xylophilus* via OPs and CBs, BxACE1 and BxACE2 showed different inhibition profiles. BxACE1 was less sensitive to the tested OPs but was more sensitive to the tested CBs than BxACE2^31^. The ACE active site gorge entrance consists of many aromatic residues, which can affect the binding affinity to substrates or inhibitors. In several cases investigated at the molecular level, the insect *ace* gene implicated in target site resistance is *ace*1^25^,^32^. However, OP resistance has been associated with mutations in both *ace*1 and *ace*2 in the wheat aphid *Sitobion avenae*^33^. Recent research also suggested that the neurological AChE is encoded by *ace*2 in a majority of Hemiptera insects^34^. In the present study, no significant difference was observed in ACE1 amino acid sequences, while 97.4% amino acid sequence identity was observed between the ACE2 of RP and SP populations. Thereafter, the differential sensitivity to fosthiazate largely reflected differences in the affinity of ACE2 between the RP and SP populations of *M. incognita*, and this sequence change in ACE2 might increase the insensitivity of ACE2 to fosthiazate. Bioassays and RNAi data indicated that the resistance largely reflected AChE insensitivity rather than increased metabolism, and mutations in AChE2 were associated with resistance to fosthiazate.

Although the functional boundary of nematode AChEs between ACE1 and ACE2 has not yet been clarified, ACE1 has been observed in synaptic transmission in *C. elegans*^35^. Previous results have also suggested that ACE2 plays critical roles in synaptic transmission and may be involved in feeding, reproduction, and other behaviours^27^. However, nematodes exhibited different species- and stage-specific patterns of ACE2 expression. ACE2 was mainly expressed in the infective juveniles of *G. pallida* and was mostly detected in the head and tail ganglion regions of *C. elegans*^36^,^37^. In *M. incognita*, ACE2 was transcribed in J2 before and after hatching in females and males, and this gene has been proposed to play a role in the contraction of the pharyngeal valve during feeding^38^. However, the RNAi-mediated gene silencing of *ace*2 has no significantly different impact on the infection of *M. incognita*, suggesting that RNAi of *ace*2 has no negative effect on nematode feeding and infection or, alternatively, the RNAi effect of *ace*2 might be compensated by *ace*1. Quantitative RT-PCR analyses showed that the *ace*1 transcription level was lower than that of *ace*2 in the RP population, which also verified the interaction of *ace*1 and *ace*2 in synaptic transmission and other motor behaviours.

Most AChEs have highly conserved structures in vertebrates and invertebrates, such as the oxyanion hole and the catalytic triad^39^. However, the replacement of amino acid residues in these conserved structures significantly changed the substrate specificity of AChEs in different species^40^,^41^,^42^. Among *B. xylophilus* AChE, 2–4 residues of the 14 aromatic residues were replaced with non-aromatic amino acids. The conversion of Phe288 to Leu was observed reducing the substrate specificity of invertebrate AChEs^40^. In the ACE2 of *Kampimodromus aberrans*, a non-synonymous G191s substitution in the AChE open reading frame reduced the sensitivity to the OP insecticide chlorpyrifos^18^. In the ACE2 of the plant bug *Apolygus lucorum*, no major differences were observed between the resistant and susceptible populations in the non-synonymous sites of the *ace*2 fragment, although an A216S difference in AChE1 was fixed in chlorpyrifos-resistant populations^19^. An equivalent mutation in the *ace* gene has previously been associated with OP resistance in several insects, including *Culex pipiens quinquefasciatus*^41^ and *Plutella xylostella*^42^. Although changes in the levels of AChE enzyme have occasionally contributed to resistance, and the same mutations differently impact OP AChE sensitivity in the plant parasitic nematode^17^, the precise role of ACE2 mutations in *M. incognita* has not been certified. To explain alternative site resistance mechanisms not yet explored in the present study, further studies focusing on the analyses of single-nucleotide polymorphism mutations that affect multiple AChE loci with additive effects in RKNs are needed.

## Materials and Methods

### *M. incognita* populations

Two populations of *M. incognita* were used in the present study. The susceptible population (SP) was collected from Daxing, Beijing and cultured on *Solanum lycopersicum* in potting soil at room temperature under a 16 h light and 8 h darkness regime, without exposure to any nematicides since 2005. The resistant population (RP) was collected from a commercial greenhouse in Daxing, Beijing in 2014, which has been treated with fosthiazate at normal dosage (a.i. 3 kg hectare^−1^) twice per year from 2007. Tomatoes had been cultivated in this greenhouse for 10 years before our experiments. In July 2014, the disease incidence was over 80%, and the galling index varied between 33 and 58. Egg masses of RP and SP were extracted from infected roots of tomato and sterilized using 1% sodium hypochlorite (NaClO), then hatched at 25 °C. Hatched juveniles were collected after 4 d and kept at 4 °C for the next experiments.

### CDNA cloning of *ace*1 and *ace*2

To search for the putative *ace* gene of *M. incognita*, gene-specific primers for *ace*1 and *ace*2 were designed from the AChE sequences (GenBank: O96529, Q71JB7) available in NCBI (Table 2). The resulting primers were used to amplify the coding sequences of the orthologous AChEs from the RP and SP populations via RT-PCR. Total RNA was extracted from second-stage juveniles (J2s) of the RP and SP populations with Invitrogen TRIzol reagent (Invitrogen, Carlsbad, CA) according to the manufacturer’s instructions. First-strand cDNA was synthesized from the total RNA using SuperScript III reverse transcriptase (Invitrogen, Carlsbad, CA). Full-length versions of *ace*1 and *ace*2 were subsequently amplified from first-strand cDNA using gene-specific primers. PCR products were cloned using a pGEM-T vector (Promega, Madison, WI) and were sequenced at Sangon (Beijing, China). The sequences were assembled and analysed using SeqMan 5.0 (DNASTAR, Inc., Madison, WI, USA). The alignments of the cDNA consensus sequences from susceptible and resistant populations were manually inspected using the MegAlign program (DNAstar, Laser gene).

### RNA interference and infection test

For knocking down the *ace*2 gene of *M. incognita* and observing the effect of *ace*2 expression on infection with the nematode, dsRNA against *ace*2 was synthesized using the Hiscribe T7 *In Vitro* Transcription Kit according to the manufacturer’s instructions (New England BioLabs) using the primers T7-iace2F and T7-iace2R (Table 2). The primers used in RNAi were designed according to regions rich in siRNA. RNAi soaking was developed according to Urwin *et al*.^43^ and Chen *et al*.^33^, with minor modifications. Freshly hatched J2s of *M. incognita* were soaked in a fluorescein isothiocyanate (FITC) solution (0.1 mg mL^−1^ FITC, 3 mg mL^−1^ spermidine, 1% resorcinol and 0.05% gelatine, adjusted with 0.25 × M9 buffer) for 6 h at 16 °C on a rotator. FITC uptake was examined under a fluorescence microscope after 6 h. For knocking down *ace*2, approximately 5,000 freshly hatched J2s were incubated in the dsRNA solution (RNAi-ace2) (2 mg mL^−1^ dsRNA, 3 mg mL^−1^ spermidine, 1% resorcinol and 0.05% gelatine, adjusted with 0.25 × M9 buffer) for 6 h at 16 °C. Control nematodes were soaked in solutions without dsRNA (non-dsRNA) or with dsRNA targeted against an reporter gene, enhanced green fluorescent protein (RNAi-egfp)^44^. For each reaction, *ca*. 800 J2s were used for RT-PCR, and *ca.* 3600 J2s were used for infection assays. The RNA interference experiments were repeated in triplicate.

*S. lycopersicum* cv. Jingpeng No. 1 was cultured in a glasshouse using autoclaved soil at room temperature. Three 1-month-old tomato seedlings were planted in a 10-cm-diameter pot and were maintained in a glasshouse at 25 °C. In total, 12 seedlings were planted in 6 tubes per treatment. Approximately 1, 200 nematodes were used in each of the three treatments (soaking buffer with *ace*2 dsRNA, soaking buffer with egfp-dsRNA or soaking buffer only) and inoculated onto the roots of tomato, followed by incubation at 25 °C. After 7 days, the roots were collected and stained using acid fuchsine^45^. Nematodes within roots were counted under the microscope. Each treatment had three replications, and the results were analysed using SPSS software with Duncan’s multiple range test.

### Transcription analyses of *ace*1 and *ace*2

Relative abundance levels of the transcripts for *ace*1 and *ace*2 gene were estimated by quantitative RT-PCR analyses. All first-strand cDNA products were stored at −80 °C and used as qRT-PCR templates. Specific primers were designed for *ace*1, *ace*2 and the *actin* gene (as an internal standard) (Table 2). The 20-μl PCR mix contained 10 μl of SYBR^®^ Select Master Mix (Life Technologies, USA), 2 μl of cDNA, and 2 μl of 10 mM forward and reverse primers and distilled water. The optimized cycling program included 1 cycle of 95 °C for 2 min, followed by 40 cycles of 95 °C for 15 s, 58 °C for 15 s, and 72 °C for 40 s, with a final product dissociation stage (Applied Biosystems 7500).The relative transcript levels for each *ace* gene in the cDNAs in different populations were quantified using the 2−^ΔΔCt^ method^46^. After the *ace*2 gene was knocked down in *M. incognita* juveniles with dsRNA, the *ace*1 and *ace*2 transcription levels in the RP were determined as described above. The expression level of the egfp-dsRNA interference gene was used as a control. For each treatment, two independent biological replicates were sampled. All qRT-PCRs were performed in triplicate.

### Nematicide bioassays

Nematicide bioassays were conducted for the preliminary screening of resistant and susceptible phenotypes using three OP pro-pesticide formulations: 96.6% fosthiazate (Ishihara Sangyo Kaisha, Ltd., Japan), 90% fenamiphos and 91% phonamiphos (Chemsky Shanghai International Co., Ltd, China). The toxicity levels (median lethal concentration, LC_50_) of the nematicides to the susceptible and resistant phenotypes of *M. incognita* were determined after immersing nematodes in 24-well microplates for 72 h. Prior to performing the toxicological procedures, pro-pesticide formulations were serially diluted with acetone solution to five concentrations. Second-stage juveniles (J2s) were hatched at room temperature (approximately 25 °C) and immersed in different nematicide dilutions in 24-well microplates; nematodes in acetone solution with a mortality below 10% were used as a control. Five hundred J2s were transferred to each well, and four replicate wells were set up for each concentration in each experiment. All resistance tests were repeated four times under the same conditions. After incubation for 72 h, 1 N NaOH was dropped into the solution to identify the alive/dead nematodes^47^. Nematode mortality was determined with the percentage of dead nematodes in each well. After the *ace*2 gene was knocked down in *M. incognita* juveniles, the toxicity of fosthiazate to the resistant and susceptible population were determined again as described above. The probe analyses were used to estimate the LC_50_ values, the 95% fiducial limits of the LC_50_ estimates, and the slopes of the mortality/concentration relationships and the standard errors (SE) of these slopes^9^.

### Enzyme activity assays

*M. incognita* J2 suspentions were centrifuged and nematode precipitates (100 μl) from each of the susceptible and resistant populations were homogenized in 1.0 ml of 20 mM Tris-HCl buffer, pH 7.5, containing 0.1 mg.L^−1^ bacitracin, 1 mM benzamidine, 5 mM EDTA, and 0.1% Triton X-100 on ice. The homogenates were centrifuged at 4 °C, 12, 000 g for 1 h, and the supernatants were collected for AChE activity assays. Homogenate extraction and the AChE activity assays were repeated four times.

AChE activity was measured using acetylthiocholine iodide (ATChI) as a substrate according to Ellman *et al*.^48^, with minor modifications. Briefly, the 400-μl reaction mixture consisted of 200 μl of enzyme preparation, 100 μl of 0.1 M sodium phosphate buffer, pH 7.2, and 100 μl of 2 mM ATChI. This reaction was incubated in a water bath for 30 min, followed by the addition of 100 μl of 10 mM 5,5′-dithio-bis-[2-nitrobenzoic acid] (DTNB), 500 μl of 4% SDS and 1 ml of 0.1 M sodium phosphate buffer, pH 7.2. The assays were incubated at room temperature (approximately 25°C) in 96-well microplates, and the absorbance was measured at 412 nm in a Multiskan MK3 microplate reader (Thermo Scientific Company).

Esterase activity was measured with a-NA according to the methods of Van Asperen^49^. Briefly, the assay reaction mixture contained 250 μl of substrate solution, containing 0.2 M sodium phosphate buffe, 10 mM a-NA and 1 mM Fast Blue RR salt, pH 6.0) and 20 μl of the enzyme solution. The assays were conducted in quadruplicate in 96-well microplates, and the absorbance was measured using a microplate reader at 450 nm for 10 min at 27 °C.

Glutathione S-transferase (GST) activity was measured with 1-chloro-2, 4-dinitrobenzene (CDNB) as a substrate according to Yang *et al*.^50^. The 210-μl reaction mixture consisted of 10 μl of the diluted enzyme solution (10-fold in 0.1 M sodium phosphate buffer, pH 7.6), 100 μl of 1.2 mM CDNB, and 100 μl of 6 mM glutathione (GSH). The absorbance was measured using a microplate reader at 340 nm for 20 min at 30 °C.

### Insensitivity of AChE to fosthiazate

To detect the insensitivity of AChE to fosthiazate, 200 μl of the AChE enzyme preparation of *M. incognita* J2 and 100 μl of 2 mM ATChI were mixed together with 100 μl of different fosthiazate dilutions (from 10^−8^ M to 10^−2^, diluted with 0.1 M sodium phosphate buffer, pH 7.2). An assay reaction mixture without the fosthiazate dilution was used as a control. The reaction mixtures were incubated in a water bath for 30 min. Subsequently, 100 μl of 10 mM DTNB, 500 μl of 4% SDS and 1 ml 0.1 M sodium phosphate buffer, pH 7.2, were added^48^. The assays were completed at room temperature in 96-well microplates, and the absorbance was measured at 412 nm using a microplate reader as described above. All of the insensitivity assays were repeated three times. The inhibition rates of different fosthiazate concentrations against AChE were analysed using the minus logarithm of concentration (−logC). After the *ace*2 gene was knocked down in *M. incognita* juveniles of the RP populations, the inhibition rates of fosthiazate against AChE in RNAi-ace2 populations and non-dsRNA populations were measured as described above. The inhibition rate of fosthiazate against AChE in RNAi-egfp populations was used as a control.

### Statistical analysis

The means and standard errors of the data obtained from the nematicide bioassay, infection experiments and transcription analyses were subjected to statistical analyses using SPSS version 21. The significant differences (P ≤ 0.05) between the treatments were determined according to Duncan’s multiple range test.

## Additional Information

**How to cite this article**: Huang, W.-K. *et al*. Mutations in Acetylcholinesterase2 (*ace*2) increase the insensitivity of acetylcholinesterase to fosthiazate in the root-knot nematode *Meloidogyne incognita*. *Sci. Rep.* **6**, 38102; doi: 10.1038/srep38102 (2016).

**Publisher's note:** Springer Nature remains neutral with regard to jurisdictional claims in published maps and institutional affiliations.

## Figures and Tables

**Table 1:** *M. incognita* infection in tomato roots after *ace*2 RNA interference.

Treatment	Number of nematodes used for inoculation	Number of penetrating nematodes	Percentage of penetrating nematodes (%)
RNAi-*ace*2	400	113.33 ± 10.01	28.33 ± 2.53a
Non-dsRNA	400	106.67 ± 17.04	26.67 ± 4.26a
RNAi-egfp	400	92.67 ± 14.98	23.17 ± 3.31a

The means in each column followed by the same letter do not significantly differ (P ≤ 0.05) according to a Duncan’s multiple range test.

**Table 2:** Primers used for cDNA amplification, dsRNA synthesis and qRT-PCR analysis.

Function	Primer	Sequence (5′–3′)
cDNA amplification	Mi-ace1-F	ATGATGGATTATTCAATAGAGGACAG
	Mi-ace1-R	CTATTTTATTCCACAAACATCATTATCACC
	Mi-ace2-F	GAGGTGAATTATGCGCAAACGAAG
	Mi-ace2-R	TTATTTGAAAATAAATGATGAATACAGGAAAGATATTTCAGG
dsRNA template synthesis	T7-iace2-F	TAATACGACTCACTATAGGGGATGGTAAATCTGTTCACAC
	T7-iace2-R	TAATACGACTCACTATAGGGGCACGCTCTTGAACCG
	T7-iegfp-F	TAATACGACTCACTATAGGGGAGTACAACTACAACAGCCAC
	T7-iegfp-R	TAATACGACTCACTATAGGGACGAACTCCAGCAGGACCAT
qRT-PCR	q-actin-F	GGGTATGGAATCTGCTGGTAT
	q-actin-R	AGAAAGGACAGTGTTGGCGTA
	q-ace2-F	GCTGGTGATGCTGTTTCTC
	q-ace2-R	CCACAAGAATGCTAACATCACG

The T7 sequences are underlined.

**Table 3:** The LC_50_ values of *M. incognita* measured for three organophosphates.

Organophosphates	Nematode populations	LC50^a^ (μg ml^−1^)	95% CL^b^	Slope ( ± SE)	RR^c^
Fosthiazate	RP	152.18	98.12–217.56	1.98 ± 0.34	2.74
	SP	55.54	38.20–86.57	1.06 ± 0.38	—
Fenamiphos	RP	13.51	8.39–20.58	2.73 ± 0.44	1.08
	SP	12.48	7.41–19.62	2.75 ± 0.39	—
Phonamiphos	RP	43.29	31.12–64.35	3.07 ± 0.31	1.05
	SP	41.26	29.58–61.46	3.12 ± 0.43	—

^a^Concentrations of nematicides causing 50% mortality at 72 h post-treatment.

^b^Confidence limit.

^c^RR (resistance ratio) = LC_50_ (the resistant population) ÷ LC_50_ (the susceptible population).

**Table 4:** The LC_50_ values of the resistant and susceptible popualtion of *M. incognita* measured for fosthiazate after RNA interference.

Populations	RNA interference	LC50^a^ (μg ml^−1^)	95% CL^b^	Slope ( ± SE)	RR^c^
Resistant population	RNAi-*ace*2	85.96	52.67–148.51	2.27 ± 0.34	0.60
	Non-RNAi	143.01	85.73–221.42	1.91 ± 0.42	1.00
	RNAi-egfp	141.05	80.64–214.35	1.93 ± 0.27	0.99
Susceptible population	RNAi-*ace*2	55.24	41.25–96.53	1.08 ± 0.29	0.96
	Non-RNAi	57.61	40.64–103.27	1.12 ± 0.43	1.00
	RNAi-egfp	56.65	35.27–93.38	1.09 ± 0.32	0.98

^a^Concentration of nematicides causing 50% mortality at 72 h post-treatment.

^b^Confidence limit.

^c^RR (resistance ratio) = LC_50_ (the RNAi population) ÷ LC_50_ (non-RNAi population).

**Table 5:** Activities of acetylcholinesterase and detoxifying enzymes in the homogenates of *Meloidogyne incognita* juveniles.

Enzyme (μmol min^−1^ mg^−1^ protein)	Substrate	Population	Enzyme activity	R/S
Acetylcholinesterase	ATChI	RP	1.65 ± 0.04 × 10^−7^	0.87
		SP	1.89 ± 0.05 × 10^−7^	—
Esterase	a-naphthyl acetate	RP	9.14 ± 0.06 × 10^−7^	1.01
		SP	9.08 ± 0.05 × 10^−7^	—
Glutathione S-transferase	CDNB	RP	63.57 ± 10.64 × 10^−7^	0.97
		SP	65.72 ± 9.89 × 10^−7^	—

R/S = Enzyme activity (RP population) ÷ Enzyme activity (SP population).
